# Delayed Emergence From Anesthesia Due to Posterior Reversible Encephalopathy Syndrome (PRES): A Case Report

**DOI:** 10.7759/cureus.71986

**Published:** 2024-10-21

**Authors:** Inês Ferraz, Sofia Carvalho, Verónica Schuler, Pedro Antunes

**Affiliations:** 1 Anesthesiology, Hospital Beatriz Ângelo, Loures, PRT; 2 Anesthesiology, Hospital da Luz Lisboa, Lisbon, PRT

**Keywords:** delayed emergence from anesthesia, hypertension, neurotoxicity, ophthalmic surgery, posterior reversible encephalopathy syndrome, postoperative blindness, vasogenic edema

## Abstract

Posterior reversible encephalopathy syndrome (PRES) is a rare clinical and radiological syndrome that presents as rapid onset of neurological symptoms such as headache, visual loss, impaired mental status, and seizure activity associated with characteristic focal white matter vasogenic edema. When promptly recognized and managed, these changes are usually reversible. PRES is most commonly associated with hypertensive crises, renal insufficiency, and the use of immunosuppressive therapies, though it may arise in various clinical contexts. Despite its significance, reports of PRES in the field of anesthesiology remain limited. This case report presents the case of a 46-year-old male admitted for elective ambulatory ophthalmic surgery under general anesthesia who developed delayed emergence from anesthesia and post-operative blindness, both attributed to the intraoperative onset of PRES.

Anesthesiologists should be vigilant for PRES as a potential complication during the perioperative period, and consider it in the differential diagnosis for delayed emergence from anesthesia. Clinical suspicion should warrant prompt imagiological confirmation by magnetic resonance imaging (MRI), as delayed recognition and management can result in severe and long-term neurological disability.

## Introduction

Posterior reversible encephalopathy syndrome (PRES) is a rare but clinically significant syndrome with distinctive radiological and neurological features. It was first described by Hinchey et al. in 1996 and was previously known as reversible posterior leukoencephalopathy syndrome, reversible posterior cerebral edema syndrome, and reversible occipital parietal encephalopathy [1]. The syndrome presents with rapid-onset neurological symptoms such as headache, visual loss, impaired mental status, and seizure activity. The severity of symptoms may vary, with impaired mental status ranging from mild confusion or agitation to coma and visual disturbances ranging from blurred vision to cortical blindness. There is a frequent but not mandatory association with acute hypertension. Neuroimaging is essential for diagnosis, with magnetic resonance imaging (MRI) being the most sensitive in showing the characteristic white matter vasogenic edema affecting mainly the posterior occipitoparietal lobes of the brain [2-4]. Although rare, there seems to be an increase in the diagnosis of this clinical syndrome, which may be related to the improvement and increased availability of brain imaging. When promptly recognized and managed, both symptoms and the changes seen in MRI usually resolve over days to weeks [2].

The etiology of PRES remains largely unknown and controversial. The most accepted theory relates the syndrome to severe hypertension leading to dysfunction of cerebral blood flow autoregulation. Cerebral blood flow is regulated by the phenomena of vasodilation and vasoconstriction, which usually manage to maintain adequate tissue perfusion while avoiding excessive intracerebral pressure. It is believed that acute uncontrolled hypertension leads to hyperperfusion and endothelial damage, resulting in interstitial extravasation of fluid, causing areas of vasogenic edema that lead to the neurological syndrome described. While this theory proves to be the most consensual among experts, it does not explain why PRES occurs in the absence of hypertension and why the extent of the edema is not directly proportional to the severity of the blood pressure (BP) elevation. An alternative theory is that PRES is a result of a systemic pro-inflammatory state leading to endothelial dysfunction. This pro-inflammatory state may be related to sepsis, auto-immune disease, preeclampsia/eclampsia, and transplantation. Further research is required to understand the etiology and physiopathology of this condition [2-6].

Although this entity is most commonly linked to hypertensive emergencies, renal insufficiency, and immunosuppressive drug use, PRES can arise in a broad spectrum of clinical scenarios, including the perioperative period. Given the multitude of etiological factors contributing to PRES, recognition of this syndrome in perioperative settings is of utmost importance; particularly in cases of delayed emergence from anesthesia. Such delayed recovery may serve as a critical indicator of underlying neurological compromise, as seen in the case discussed herein. Reports of PRES occurring in the context of anesthesia and surgery remain scarce, further emphasizing the importance of heightened awareness among anesthesiologists when managing patients with significant comorbidities [7-9]. This case report presents the case of a 46-year-old male who underwent elective ophthalmic surgery under general anesthesia. Despite an initially uneventful procedure, the patient experienced delayed emergence from anesthesia and post-operative cortical blindness, later attributed to the intraoperative onset of PRES. This case underscores the necessity of including PRES in the differential diagnosis of delayed recovery from anesthesia as prompt recognition and treatment are critical to preventing severe long-term neurological sequelae.

## Case presentation

A 46-year-old male was admitted to our hospital for elective silicone oil extraction from the right eye, following a vitrectomy performed one month earlier due to proliferative diabetic retinopathy. The surgery was proposed to be performed under general anesthesia and discharge from the hospital was planned for the same day. His medical history included type I diabetes mellitus, poorly controlled with biphasic isophane insulin suspension, and essential hypertension treated with an angiotensin-converting enzyme (ACE) inhibitor. He had previously undergone three general anesthesias without any reported complications; the last of which was performed just one month before for right eye vitrectomy and phacoemulsification with intraocular lens placement. He had no history of allergies, and his functional residual capacity was maintained at >4 METS (Metabolic Equivalent of Task). On physical examination, he had a body mass index of 22.8 kg/m² and presented no signs of a difficult airway. His blood analysis revealed anemia, and poorly controlled diabetes, as shown in Table 1. Otherwise, his physical examination and tests, including blood electrolytes, renal and liver function tests, and electrocardiogram, were normal. No neurological deficits were observed.

**Table 1:** Altered pre-operative blood test findings

	Patient result	Normal range
Hemoglobin (g/dL)	12.2	13.0-17.0
Blood glucose (mg/dL)	238	60-100
HbA1c (%)	11	4.3-5.9

On the day of the surgery, the patient took his routine antihypertensive medication, but bypassed his insulin treatment. On admission to the hospital, his BP was 190/100 mmHg. Given the elevated BP, he was given oral nifedipine (calcium channel blocker) and intravenous (IV) midazolam for anxiolysis. The re-evaluation before transfer to the operating room (OR) was 139/80 mmHg.

In the OR, the patient was monitored with standard ASA monitoring and anesthesia depth monitor (bispectral index, BIS®). General anesthesia was induced using IV fentanyl 0.15 mg and propofol 200 mg; muscle relaxation was achieved with rocuronium 50 mg. Orotracheal intubation with a 7.5 orotracheal tube was performed without difficulty. Ventilation was adjusted to maintain EtCO₂ at 30-35 mmHg. Anesthesia was maintained with desflurane, fentanyl (0.05 mg), and intermittent boluses of rocuronium (30 mg). The surgery lasted four hours. Due to hyperglycemic values (capillary glycemia 230 mg/dL), insulin treatment was administered intra-operatively (IV actrapid 8 UI); with adequate control (capillary glycemia 141 mg/dL). Throughout the procedure, the patient presented with hemodynamic lability (57 < mean arterial pressure (MAP) < 97 mmHg), with capnography and oximetry remaining unaltered. No vasoactive drugs were administered.

At the end of the procedure, neuromuscular blockade was reversed with sugammadex 200 mg, and spontaneous ventilation was restored, and adequate. Consciousness was not recovered despite normalization of end-tidal desflurane levels. The patient presented with sudoresis, hypertension (BP 161/90 mmHg), and sinus tachycardia (heart rate 110 bpm). On neurological evaluation, the left pupil was unreactive (interpretation of such finding was made difficult by the fact that this eye had also undergone surgery a few months previously). Blood glycemia levels were 131 mg/dL; the auricular temperature was 36ºC. Blood gas analysis showed no alterations. Both naloxone (0.4 mg + 0.4 mg) and flumazenil (0.2 mg) were administered, with no change.

Immediate brain computed tomography (CT) revealed no acute abnormalities. The patient was transferred under sedation and mechanical ventilation, to the Intensive Care Unit (ICU), and shortly after arrival, he experienced a generalized tonic-clonic seizure, treated successfully with diazepam 10 mg IV. Decerebration movements were present in the upper right limb. Hypertension treatment was implemented with a target MAP of 90 mmHg.

**Figure 1:**
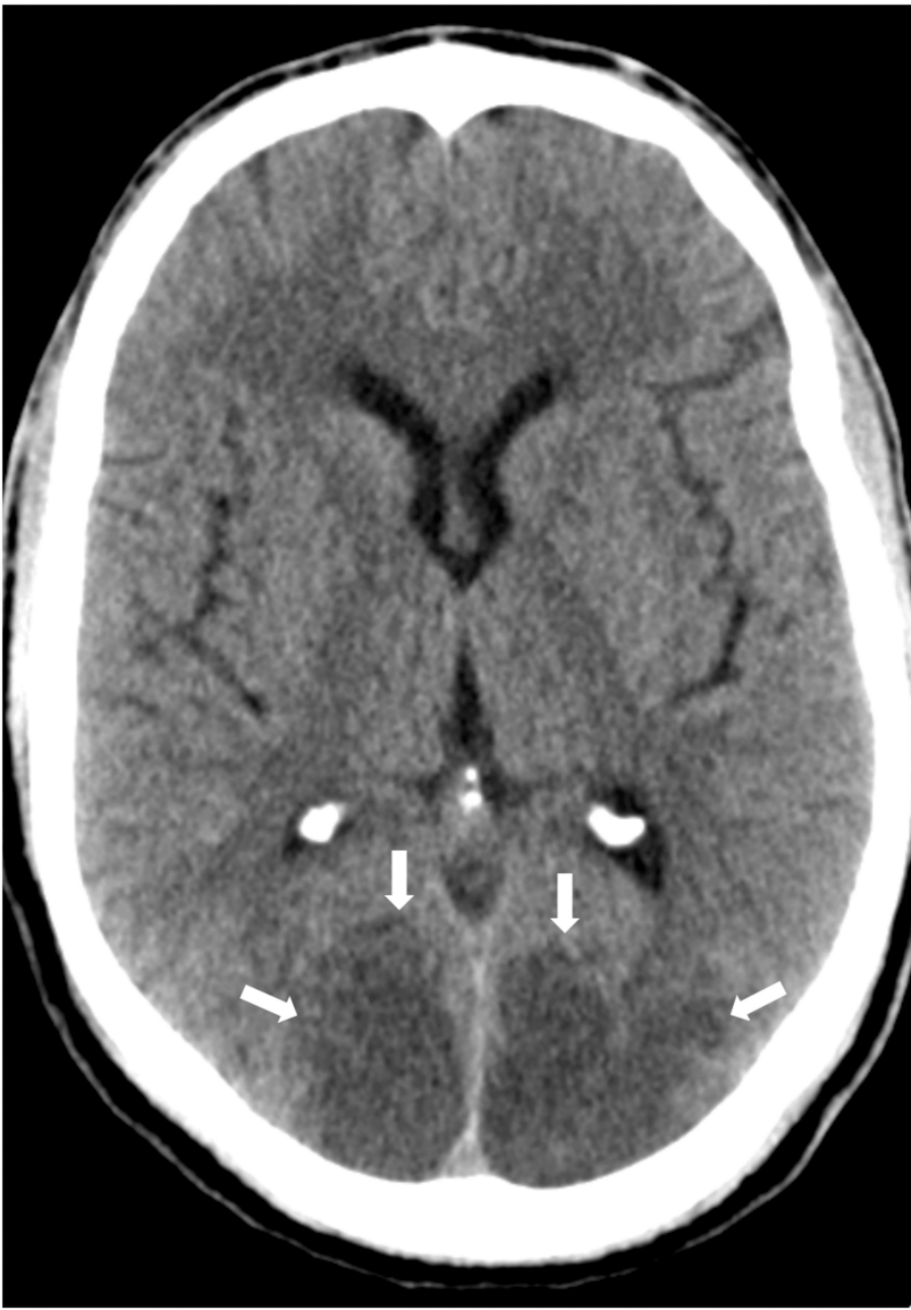
Brain CT scan showing parieto-occipital areas of cortico-subcortical edema CT: computed tomography

**Figure 2:**
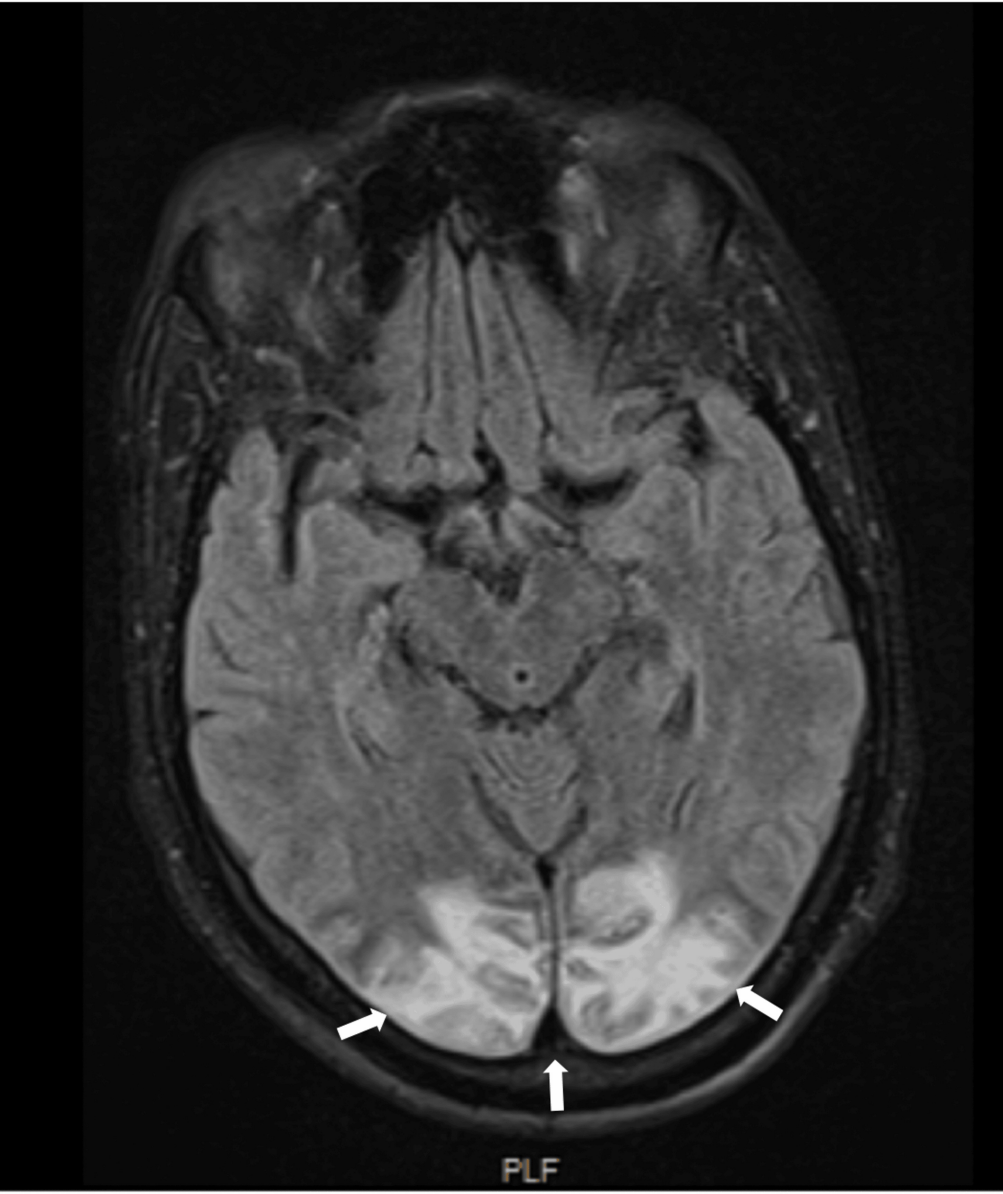
FLAIR sequence brain MRI, of the patient on postoperative day 5, showing areas of vasogenic edema in the parieto-occipital lobes FLAIR: Fluid-attenuated inversion recovery; MRI: Magnetic resonance imaging

On the first postoperative day, sedation was suspended, and the patient regained consciousness, but exhibited bilateral cortical blindness and right-sided hemiparesis. A follow-up brain CT scan (Figure 1) revealed extensive bilateral cortico-subcortical edema within the parieto-occipital lobes, consistent with PRES. Brain MRI (Figure 2) confirmed the presence of vasogenic edema, with petechial hemorrhages, further corroborating the diagnosis.

By postoperative day 5, BP and glycemic control had been accomplished, and motor function on the right side had improved, though the patient’s visual deficits persisted. The patient was discharged from the ICU, having recovered partially, with persistent blindness in the left eye and light perception only in the right eye. A panoply of diagnostic exams was performed, such as echocardiogram, Doppler of neck blood vessels, and electroencephalogram. All of these exams were found to be without pathological findings. The patient was discharged from the hospital on day 13 postoperative with the following deficits: complete blindness of the left eye, light perception in the right eye, and negative menace reflex bilaterally. On a six-month follow-up, he maintained the same deficits.

## Discussion

The onset of PRES in this patient following elective ophthalmic surgery underlines the interplay of multiple factors, including his pre-existing comorbidities, poorly controlled diabetes and hypertension, and the hemodynamic instability and metabolic derangements in the intra-operative period, which likely exacerbated the risk of developing PRES. The pathophysiology of PRES involves a breakdown of cerebral autoregulation and endothelial dysfunction, with rapid increases in BP overwhelming the blood-brain barrier. This results in vasogenic edema, predominantly in the posterior circulation of the brain [10]. Hyperglycemia contributes to microvascular injury and further impairs autoregulation [1,3]. The hypertensive crisis in the perioperative period likely triggered the onset of vasogenic edema in this case.

Delayed emergence from anesthesia and PRES

Delayed emergence from anesthesia is a critical event in the perioperative period, defined as the failure to regain consciousness within the expected timeframe following cessation of anesthetic agents. This phenomenon, while often multifactorial, raises significant concerns about underlying neurological complications, particularly in patients with risk factors, such as hypertension, metabolic disturbances, or other comorbidities. In this case, the patient experienced delayed emergence, which subsequently revealed an underlying case of PRES.

Delayed emergence can be broadly categorized into two types: pharmacological and pathophysiological. Pharmacological causes include prolonged action of anesthetic agents due to overdose, drug interactions, or impaired clearance. This is particularly concerning in patients with hepatic or renal dysfunction. However, in this patient, preoperative liver and kidney function tests were normal, and desflurane - an inhalational anesthetic with a rapid elimination profile - was used, reducing the likelihood of a pharmacokinetic issue.

**Figure 3:**
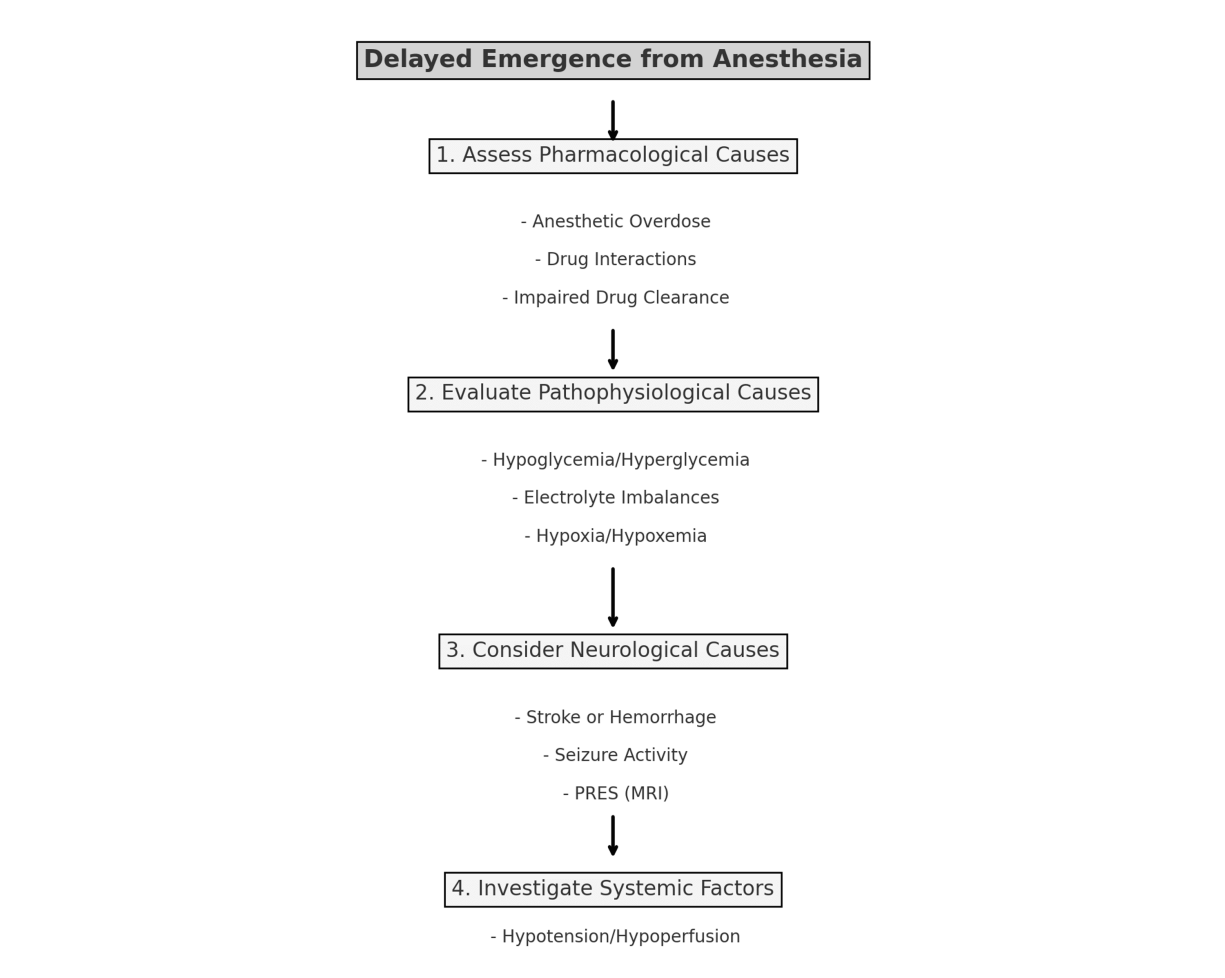
Differential diagnosis algorithm for delayed emergence from anesthesia PRES: Posterior reversible encephalopathy syndrome; MRI: Magnetic resonance imaging

Pathophysiological causes, on the other hand, involve disruptions in normal brain function. These may include metabolic disturbances such as hypoglycemia, hyperglycemia, electrolyte imbalances (particularly sodium and calcium), or even hypoxia during surgery. However, when metabolic or systemic causes are excluded, as in this case where glucose and electrolyte levels were within the normal range, delayed emergence strongly suggests an underlying neurological process [11]. A differential diagnosis algorithm for delayed emergence from anesthesia is presented in Figure 3.

In the case of PRES, the delayed emergence likely resulted from widespread cortical dysfunction secondary to vasogenic edema. The cerebral cortex, particularly the parieto-occipital regions, typically affected in PRES, plays a critical role in maintaining consciousness and wakefulness. The diffuse nature of cerebral involvement in PRES, evidenced by MRI findings of extensive bilateral vasogenic edema, disrupts normal neuronal activity and delays the restoration of consciousness [3].

Another aspect to consider is the potential for cerebral hypoxia or altered perfusion due to intraoperative BP fluctuations. Although the patient remained hemodynamically stable during the procedure, periods of low MAP could have contributed to transient ischemia, exacerbating the development of edema. PRES often manifests following hypertensive crises, but in some patients, fluctuations in BP - particularly drops in MAP - can aggravate the underlying endothelial dysfunction, worsening cerebral edema [6].

PRES in the perioperative setting (diagnostic and therapeutic considerations)

PRES is relatively rare in the perioperative context, but its incidence is likely underreported due to a lack of recognition [7,9]. Given the high stakes of anesthesia-induced altered consciousness, delayed emergence should trigger an immediate evaluation for neurological conditions such as PRES, particularly in patients with predisposing factors like hypertension, renal insufficiency, or metabolic dysregulation, when exposed to perioperative stress and anesthesia-induced hemodynamic fluctuations. In the case presented, delayed emergence was the first clinical indication of PRES, which was later confirmed with neuroimaging.

The clinical presentation of PRES in the perioperative setting is often subtle, starting with mild neurological symptoms, such as confusion, or disorientation, which may be mistaken for residual anesthetic effects [9]. However, the progression to more severe manifestations, including seizures, cortical blindness, and motor deficits, necessitates an urgent diagnostic workup. In this patient, the initial delay in recovery from anesthesia was followed by seizures and cortical blindness, providing strong clinical clues toward a neurological etiology.

The timing of neuroimaging is critical in such cases. While the initial brain CT scan in this patient showed no acute abnormalities, MRI is far more sensitive for detecting the vasogenic edema characteristic of PRES. Sequences like fluid-attenuated inversion recovery (FLAIR) are particularly effective in identifying these changes in the posterior brain regions, where PRES typically manifests [5,8,10].

The early recognition of delayed emergence as a potential sign of PRES is crucial, as delayed diagnosis may result in significant morbidity, including permanent neurological deficits. In this case, the prompt transfer to the ICU and initiation of antihypertensive therapy likely prevented further neurological deterioration, although the patient did suffer persistent visual deficits. Early intervention with appropriate BP control, seizure management, and supportive care are key to reversing the vasogenic edema associated with PRES, as there are no known specific therapeutic options [2,3].

Given the potential for permanent disability, if PRES is not recognized and treated early, anesthesiologists must remain vigilant for signs of delayed emergence, particularly in patients with risk factors. The clinical hallmark of delayed emergence, in combination with other neurological symptoms such as visual disturbances or seizures, should prompt immediate neuroimaging and aggressive management to mitigate long-term damage.

Implications for anesthesiologists

This case underscores the importance of anesthesiologists maintaining a high index of suspicion for PRES, particularly in patients with known risk factors, such as poorly controlled diabetes and hypertension. Delayed emergence from anesthesia should prompt a thorough assessment for possible neurological complications, including PRES [9,11]. Prompt recognition and intervention are crucial in preventing long-term sequelae such as permanent visual loss, as seen in this patient.

## Conclusions

PRES is a rare but significant complication in the perioperative setting, particularly in patients with predisposing conditions such as poorly controlled diabetes and hypertension. Anesthesiologists must consider PRES in the differential diagnosis of delayed emergence from anesthesia, as timely diagnosis and management are crucial in preventing irreversible neurological damage. In this case, early recognition and aggressive management of BP were key in mitigating the severity of the patient's condition, although permanent visual impairment persisted. This case emphasizes the need for heightened awareness of PRES as a potential perioperative complication in at-risk patients.
